# How to fall into a new routine: factors influencing the implementation of an admission and discharge programme in hospitals and general practices

**DOI:** 10.1186/s12913-022-08644-5

**Published:** 2022-10-25

**Authors:** Johanna Forstner, Nicola Litke, Aline Weis, Cornelia Straßner, Joachim Szecsenyi, Michel Wensing

**Affiliations:** Department of General Practice and Health Services Research, Heidelberg University Hospital, Im Neuenheimer Feld 130.3, Marsilius Arkaden, Turm West, D-69120 Heidelberg, Heidelberg, 69120 Germany

**Keywords:** Implementation, Admission management, Discharge management, Hospital, General practice, Routine, Middle management

## Abstract

**Introduction:**

The VESPEERA programme is a multifaceted programme to enhance information transfer between general practice and hospital across the process of hospital admission, stay and discharge. It was implemented in 7 hospitals and 72 general practices in Southern Germany. Uptake was heterogeneous and overall low. A process evaluation aimed at identifying factors associated with the implementation of the VESPEERA programme.

**Methods:**

This was a qualitative study using semi-structured interviews in a purposeful sample of health workers in hospitals and general practices in the VESPEERA programme. Qualitative framework analysis using the Consolidated Framework for Implementation Research was performed and revealed the topic of previous and new routines to be protruding. Inductive content analysis was used for in-depth examination of stages in the process of staying in a previous or falling into a new routines.

**Results:**

Thirty-six interviews were conducted with 17 participants from general practices and 19 participants from hospitals. The interviewees were in different stages of the implementation process at the time of the interviews. Four stages were identified: Stage 1,’Previous routine and tension for change’, describes the situation in which VESPEERA was to be implemented and the factors leading to the decision to participate. In stage 2,’Adoption of the VESPEERA programme’, factors that influenced whether individuals decided to employ the innovation are relevant. Stage 3 comprises ‘Determinants for falling into and staying in the new VESPEERA-routine’ relates to actual implementation and finally, in stage 4, the participants reflect on the success of the implementation.

**Conclusions:**

The individuals and organisations participating in the VESPEERA programme were in different stages of a process from the previous to the new routine, which were characterised by different determinants of implementation. In all stages, organisational factors were main determinants of implementation, but different factors emerged in different implementation stages. A low distinction between decision-making power and executive, as well as available resources, were beneficial for the implementation of the innovation.

**Trial registration:**

DRKS00015183 on DRKS / Universal Trial Number (UTN): U1111-1218–0992.

## Introduction

Care transitions are challenging and can pose a threat to continuity of care and patient safety [1, 2]. A very important care transition is that from hospital to home, which, if not well managed, can lead to unplanned readmission to hospital. There are a number of studies aimed at improving health care at this interface, but the evidence is inconclusive. In addition, the process of hospital admission, i.e. the transition from home to the hospital, is not well researched [3–6]. The VESPEERA programme for hospital admissions and discharges (VESPEERA—Improving care across sectors: An admission and discharge model in general practice and hospitals) addressed this issue by involving primary care teams in a multifaceted intervention to improve information transfers across the whole process from admission by general practice through hospitalisation and discharge to follow-up care by general practice. The VESPEERA programme was implemented in 7 hospitals and 72 general practices in a defined region in Southern Germany [7].

The implementation of innovations to improve care transitions is complex. These innovations do not only consist of several components [8]. They often target different types of organisations that differ in their structural characteristics, legal regulations and many other contextual factors [9]. In addition to the characteristics of the innovation itself, the implementation and the context in which the innovation is implemented in are prerequisites for the successful implementation of innovations [10]. In the case of VESPEERA, the target organisations were general practices participating in a general practitioner (GP)-based care programme and hospitals without further inclusion criteria.

The literature describes numerous factors that influence the implementation of innovations in primary care, inpatient care or care transitions. In a systematic review of systematic reviews, Lau et al. [11] report resources, the fit with daily practice, roles and competencies of individuals and ‘ease of use and adaptability to local circumstances’ as determinants of achieving change in primary care [11]. Geerligs et al. [12] describe barriers to implementation in a hospital setting at the system, staff and intervention levels, which also interact with each other. They describe determinants for implementation such as staff workload and turnover, fit of the intervention with hospital IT, the need to prioritise between daily work and innovation and the possibility of integration into existing processes. In addition, they point to the importance of training to raise awareness of the interventions and their benefits for the stakeholders involved [12]. In relation to cross-sectoral innovations, discharge planning or care transitions, several studies have found that the following factors are crucial for implementation: Clarity of roles and responsibilities [13, 14], evidence base and perceived benefits, although it often takes time for these benefits to become visible/ recognisable [14–16], champions or leaders who commit to the innovation over a long period of time and stick with it even when others stop [14, 15, 17], low additional workload due to the innovation [16] and high additional workload for study-related activities such as obtaining informed consent from study participants [14]. Resources have both been described as constraining (when there is not enough staff or time) [16], but lack of resources can also facilitate change when staff recognise that they need to find a way to work more efficiently [17]. In addition, high organisational fit can facilitate implementation [14].

In evaluating the effectiveness of the VESPEERA programme, the targeted sample size was not achieved [19]. In addition, the number of patients included by each practice varied widely, with some unable to include any patients at all. Hospitals were unable to identify VESPEERA patients and therefore could not use the intervention. In addition, intervention fidelity (delivery of the intervention components as planned [18]) in VESPEERA was heterogeneous and overall low.

The implementation of the complex VESPEERA programme was therefore complemented by a process evaluation. The aim of this study was to identify factors that were associated with the implementation of the VESPEERA programme and that thus led to the heterogeneous and overall suboptimal uptake of the VESPEERA programme in hospitals and general practices.

## Methods

### Design and setting

This qualitative observational study was part of the evaluation of the VESPEERA study. The VESPEERA study aims to improve communication between hospitals and general practices and thereby improving patient’s continuity of care. In the German healthcare system, patients are referred to hospital by ambulatory physicians for planned admissions, while others enter hospital as (unplanned) emergency admissions. Both should be included in the VESPEERA programme. Patients who had no contact with their GP before admission were included after hospital discharge.

**Fig. 1:**
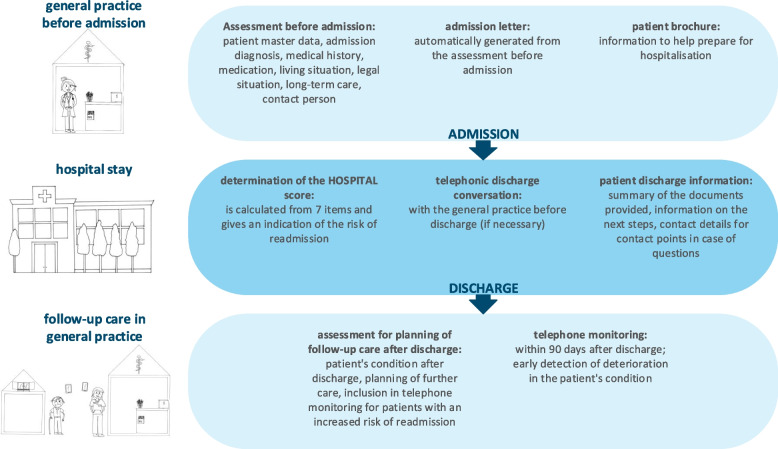
Components of the VESPEERA programme [21]

The programme includes pre-hospital, pre-discharge and post-discharge components. The components are shown in Fig. 1. A detailed description of the VESPEERA components can be found elsewhere [7, 19]. These components were implemented through a series of strategies, which are summarised in Table 1. A detailed description of the implementation strategies can be found in the study protocol [20].

**Table 1:** Implementation strategies

Strategies	Description
Consensus discussions	Involvement of representatives of all stakeholders (hospitals, general practitioners, health insurers, patients) in the development of the intervention components with the aim of reaching consensus.
Formal commitments	Adaptability was promoted in terms of integrating the intervention components into the hospitals; hospitals had to describe this in a formal commitment form.
Change of record system	The intervention components in general practices were supported by a software tool called “CareCockpit”, which had already been used previously for general practice-based case management.
Train-the-trainer	General practice staff were trained in the use of the CareCockpit software and study measures by teams of trainers who were themselves trained by the study central office.
Educational materials	Hospitals and general practices received educational material on the intervention components and study conduction, such as investigator site files or video tutorials.
Ongoing consultation	General practices and hospitals were continuously supported in the implementation of the intervention by the study central office through updates by mail and post, telephone calls and refresher training for general practices.
Provision of feedback	The hospitals and general practices were offered feedback in the form of three benchmarking reports and feedback meetings in the form of workshops, which included first results from the evaluations.
Financial incentives	Incentives were created for service providers in that they received fee-for-fee remuneration for the provision of the intervention components.

The VESPEERA programme was applied from May 2018 until the end of September 2019. The aim of the process evaluation was to examine intervention fidelity, perceived effects, working mechanisms, feasibility and contextual factors influencing the implementation [20]. Ethical approval was obtained by the Ethics Committee of the Medical Faculty Heidelberg (S-352/2018) for the process evaluation of the VESPEERA study. All participants gave their written informed consent prior to the interview. The study was registered prior to data collection (DRKS00015183 on DRKS / Universal Trial Number (UTN): U1111-1218–0992).

The study was documented according to the Consolidated Criteria for Reporting Qualitative Studies (COREQ) checklist [22].

### Study sample

The purposeful sample consisted of hospital management such as quality managers and ward managers (who had to work in a participating hospital department or to be involved in the implementation of the VESPEERA programme on a higher hierarchical level), physician and nursing staff in the participating hospitals as well as GPs and Care Assistants in General Practice (VERAH, German: *Versorgungsassistentin in der Hausarztpraxis*) who have committed to implementing the VESPEERA programme. VERAHs are medical assistants with further training in case management [23]. They were actively involved in the implementation of the VESPEERA programme and conducted assessments and telephone monitoring with patients in the CareCockpit software. All participants had to be at least 18 years old, had to be able to speak and write German and had to be able to give their informed consent to participate in the study.

The recruitment of staff in general practices and hospitals took place as planned. All participating GPs and VERAHs were contacted with a postal invitation letter. Hospital staff was recruited by contacting the contact person for all project-related communication, who was asked to forward the interview invitation to all eligible persons [20]. All persons who expressed interest in participating were called by the study central office and informed about study participation. Non-participation was not documented. None of the participants dropped out of the study.

### Data collection and measures

Qualitative data was collected in interviews using a self-developed semi-structured interview guide. The guide was developed using the Interview Guide Tool of the Consolidated Framework for Implementation Research (CFIR) by Damschroder et al. [24]. The interview guide focused on the VESPEERA programme and its implementation. The following topics were addressed: current measures of admission and discharge management, effects of the new legal regulation on discharge management (German: *Rahmenvertrag Entlassmanagement*) on internal processes and cooperation with other health care providers as well as other aspects of the hospital admission and discharge process, implementation of the VESPEERA programme, perceived effects of the programme as well as determinants of implementation. The interview guide was pilot tested before the first interview, no adjustments to the interview guide were made during data collection.

The interviews were conducted from September 2018 through July 2020 by three female researchers and doctoral candidates from the Department of General Practice and Health Services Research at Heidelberg University Hospital, who were approx. 30 years of age at the time of data collection. JF has a background in health sciences, health services research and implementation science, NL has a background in speech and language therapy, interprofessional health care, health services research and implementation science and AW has a background in social sciences and medical process management. The interviewees were informed that the aim of the interviews was to gain insight into their experiences with admission and discharge management and with the VESPEERA programme. JF knew some of the interview partners from the VESPEERA intervention phase. Therefore, in cases where the contact between JF and the interviewees was close, the interviews were conducted by NL or AW.

The interviews were conducted either as telephone interviews or face-to-face interviews (in private at the participants’ workspace), depending on the participants’ preference. All interviews were audio recorded and hand-written notes were taken. Interviews were not repeated and transcripts were not returned to the participants for correction. Interviews were transcribed verbatim using simplified transcription rules, omitting dialect or informal language/ slang.

Socio-demographic information on age, gender, profession, years of work and structural characteristics of the organisation in which the participants was collected using a paper questionnaire. The inclusion of interviews in the analysis was completed with the achieved saturation of codes and content in the analysis of interview data.

### Data analysis

First, qualitative framework analysis was conducted by a multi-professional research team (JF, NL, AW, CS – female, about 30 years of age, GP and health services researcher) using the CFIR [24] to sort the data and enable the identification of more specific aspects.

In a second step, JF and NL met and searched all CFIR themes and subthemes for codes that specifically related to the implementation process of the VESPEERA programme. In this second step, the theme of previous and new routines emerged. Organisational routines are defined as ‘recurrent interaction patterns’ [25] (p. 645) and contribute to organisational capabilities and organisational change as they enable coordination, provide stability, allow for the unconscious execution of tasks and contribute to knowledge management [25].

For this reason, an additional keyword for ‘everyday life’,’routine’, ‘implement*’, and ‘adopt’ (all searched for using the German equivalents) was conducted in all transcripts to detect related passages in the transcripts. This was to ensure that all corresponding passages and codes were actually included.

In a third step, inductive content analysis was conducted by both authors for all of the identified codes. During this process, an inductive set of four stages was developed to describe the process of staying in a previous routine or falling into a new routine through the implementation of the VESPEERA programme. After an intercoder analysis, JF and NL met and discussed any codes that did not agree until agreement was reached.

MAXQDA versions 18 and 20 (Verbi GmbH) were used for data coding and IMB SPSS Statistics version 26 was used to analyse the socio-demographic data.

## Results

A total of 36 persons were included in this interview study. Of these, 10 were hospital managers, 3 hospital physicians, 6 hospital nurses, 6 GPs and 11 VERAHs. Table 2 gives an overview of the distribution of participants among the different groups. The duration of the interviews was 42 min on average and varied between 21 and 78 min. The participants were predominantly female (69%) and had a mean age of 45 years. Participants had a mean work experience of 15 years in their current general practice or specialty. Table 3 gives an overview of socio-demographic characteristics of the study population.

**Table 2:** Numbers of qualitative interviews conducted

	*n*
hospital staff	
management	10
physicians	3
nursing staff	6
staff in general practices	
GPs^a^	6
VERAHs^b^	11

in total36

^a^ GP = general practitioner, ^b^ VERAH = Care Assistant in General Practice (*Versorgungsassistentin in der *Hausarztpraxis)

**Table 3:** Sociodemographic characteristics of the study population

	general practices		hospitals			total
	GPs^a^	VERAHs^b^	management	physicians	nursing staff	
age	58 (50–64)^c ^*, n* = 6	40 (31–54), *n* = 11	48 (29–60), *n* = 10	50 (34–58), *n* = 3	35 (21–52), *n* = 6	45 (21–64), *n* = 36
male gender	2 (33)^d^, *n* = 6	0 (0%), *n* = 11	5 (50%), *n* = 10	3 (100%), *n* = 3	1 (17%), *n* = 6	11 (31%), *n* = 36
urban area	3 (50%), *n* = 6	6 (60%), *n* = 10	9 (90%), *n* = 10	2 (66%), *n* = 3	6 (100%), *n* = 6	26 (74%), *n* = 35
years of experience^e^	16.5 (2–25), *n* = 6	17.5 (2–38), n = 10	10 (2–18), *n* = 10	23 (7–32), *n* = 3	12 (2–28), *n* = 6	15 (2–38), *n* = 35
single practice	4 (66%), *n* = 6	5 (50%), *n* = 10				9 (56%), *n* = 16
practice size (patients per quarter year)	1467 (850–2400), *n* = 6	1775 (999–3000), *n* = 8				1643 (850–3000), *n* = 14
hospital size: basic and regular care			3 (33%), *n* = 10	1 (33%), *n* = 3	2 (33%), *n* = 6	6 (32%), *n* = 19

^a^ GP = general practitioner, ^b^ VERAH = Care Assistant in General Practice (*Versorgungsassistentin in der Hausarztpraxis*), ^c^ mean (min–max); ^d^ Frequencies (percent); ^e^ general practices: in their current practice, hospitals: in this field

**Fig. 2:**
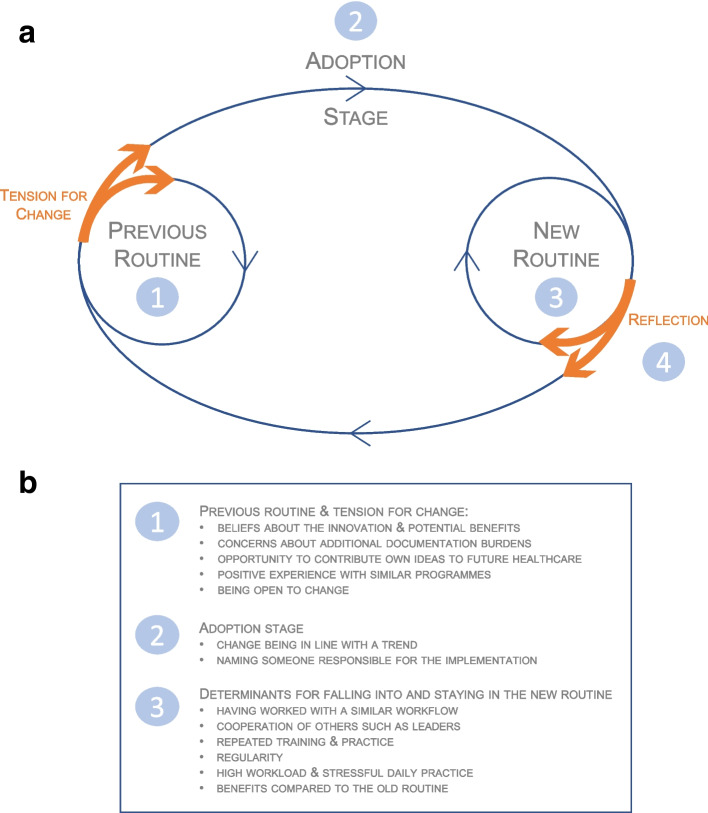
Stages of the implementation process and its’ determinants of the VESPEERA programme

The results show four stages in the process of implementing the VESPEERA programme (see Fig. 2): 1) Previous routine and tension for change (the ‘degree to which stakeholders perceive the current situation as intolerable or needing change’) [24], 2) Adoption of the VESPEERA programme (adoption in the sense of applying an innovation, also referred to as uptake, [18] or intervention fidelity), 3) Determinants of falling into and staying in the new VESPEERA-routine, and 4) Reflection: the participants’ conclusion. First, the previous routine and a tension for change describe the initial setting in which VESPEERA should be implemented. After the decision to participate in the VESPEERA programme in order to overcome the previous routine and meet the tension for change, the VESPEERA programme then had to be applied by the individuals (adoption stage). Finally, there are several factors that determine whether the new routine can be picked up and implemented. Finally, conclusions can be drawn about the success of the implementation. These four stages are described below.

### Previous routine and tension for change

VESPEERA was introduced in hospitals and general practices as an innovation, which meant a change in previous care routines. The following section describes the tension for change of the participants and other factors that had an influence on the individual decision to participate in VESPEERA and start implementing it.

In hospitals, the decision to participate mostly was up to the head physicians and administrative managers. In the case of general practices, on the other hand, there were practices where the decision was made by the GP as well as those where the VERAHs were directly involved in deciding whether the general practice would participate in the VESPEEERA programme. Furthermore, there were even practices where VERAHs were the ones who learned about VESPEERA, thought that their patients could benefit from it, and then decided to participate in the study together with the GP.

Beliefs about the innovation and the expected benefits from the innovation were described as central in the process of decision-making for initiating the implementation of VESPEERA. This type of cost–benefit consideration was also described as crucial for recruiting participating colleagues within an organisation:’Yes, and if this point ‘What’s in it for me’ comes up short in the presentation, then of course I’ve lost my audience.’ (nurse). However, in some practices, GPs indicated that they were already satisfied with the existing care processes and described that they did not expect ‘any significant changes’ (GP) in their routine as a result of VESPEERA. For these participants, the decision to implement VESPEERA was mostly based on personal motivation, interest in scientific work or the possibility of creating the change.

Concerns about the additional documentation burden were mentioned as another obstacle to the decision to participate. On the other hand, most participants were motivated. Participation in VESPEERA was also described as an opportunity to influence the intervention components and the associated workloads. Participants from hospital management referred to new the legal regulation to improve discharge management *(*German: *Rahmenvertrag Entlassmanagement*), which had to be implemented around the same time. Many participants were of the opinion that the contents were not suitable to address the deficits in health care. Therefore, with their participation in VESPEERA, they wanted to take the opportunity to contribute to the design of future care based on their experience.

In general, VESPEERA was expected to address relevant problems that participants saw in relation to care transitions, namely insufficient communication between hospitals and general practices, the lack of discharge letters and information about the hospital stay and the patient’s care process: ‘And - it… well, it just doesn't work the way it should and that’s always bothered me and I actually thought it was good that it just got sorted out and then we just wanted to be there and make sure that it works well from our side as well.’ (VERAH).

In addition to positive beliefs about VESPEERA, positive experiences with similar programs (such as the PraCMan software for GP-based case management), to which VESPEERA represents a further development, were also seen as motivation for participating in VESPEERA. Participants described themselves as open to change and wanting to learn about the new programme before assessing it, as they observed among some of their colleagues.’So, on the one hand, among those in white coats – I would call it that – there is too much discussion about whether it is necessary or not, instead of just doing it and then seeing if it is useful before we start talking about it.’ (nurse). In general, the interviewees were motivated, open to innovation, self-critical and reflective about their own behaviour and strive for improvement: ‘I also find it nice to question and evaluate existing structures or activities.’ (nurse). Financial incentives were described by GPs as not relevant for participating in VESPEERA, furthermore one participant described being ‘stuck in a routine’ (GP) from which there is no way out, even if there is an external motivation such as high financial incentives. Motivating decision-makers to encourage and remind others of the new programme was described as even more helpful in starting the implementation process. Finally, one participant predicted that implementing specific VESPEERA components would be easier in smaller hospitals than in larger ones.

### Adoption of the VESPEERA programme

The adoption stage was described very heterogeneously and seems to depend on the respective hospital or practice. One of the facilitating factors is that the participants described that it is easier for them to implement VESPEERA if the change is in line with a certain trend: ‘So because actually much was already planned beforehand or was already in progress, which already worked relatively well.’ (hospital management). Furthermore, some of the participants, such as hospital management, were involved in the development of the VESPEERA programme and mentioned this as a positive factor for further implementation, as they were able to influence the fit of the programme and the organisation as well as its feasibility.

Other participants, especially frontline workers in hospitals, described that the person who decided to participate in the study or the person who was responsible for the actual implementation from middle management (e.g. team leaders) simply told them that they would be part of the study from now on. These participants were found to lack knowledge about the VESPEERA components. Some hospital staff described their implementation style as’learning by doing’ as they planned to implement the intervention without preparation as soon as a patient was identified. One participant justified their decision to use this type of implementation process based on previous experiences. The participant described that colleagues refused to accept new care routines if it seemed like a ‘dictation of further actions they had to follow from now on’ (nurse).

The progress of the implementation process was described as depending on the motivation and commitment of the individual participants. It was described as beneficial if specific individuals, such as frontline workers, were named responsible for implementing the intervention. However, some hospitals described a lack of staff, such that no one was responsible for implementing the VESPEERA components, such as identifying incoming VESPEERA patients. Consequently, some hospitals described that ‘We don't really have a… description of the process because we really don't know whose lap to drop it in’ (hospital management). Although all hospitals submitted their commitment form with a description of implementation, some hospitals described that they would start implementing the VESPEERA components as soon as the first patient entered their hospital. However, it was found that none of the hospitals had a VESPEERA patient identified. Therefore, in some hospitals, a detailed plan for the implementation of the intervention was never prepared, which may also have affected the quality of patient identification. Furthermore, it remains unclear to whom the plan described in the commitment form was communicated due to a lack of responsible staff.

In general practices, VERAHs were primarily named as responsible for implementing and executing the VESPEERA components, and responsibilities were clearly communicated. In contrast to hospitals, most participants from general practices said they had a structure or plan for how VESPEERA is implemented: ‘We have the programme, we have the roadmap, everything else is running, it’s not a problem. The staff just have to be made available.’ (GP).

As described, some GPs pointed out that they did not expect major changes through the implementation of the VESPEERA components, as there were already similar efforts and changes in practice itself. For the adoption process, this was described as beneficial, as the VESPEERA components blended into and enhanced existing processes.

### Determinants for falling into and staying in the new routine

After the adoption of the VESPEERA programme, participants reported numerous determinants that were crucial to falling into and staying in a new routine in order to implement the intervention. Falling into a routine is perceived as easier if the participant has already worked with a programme/ process that is similar to the innovation/ change and the difference between old and new is only slight. For many general practices, the change was not completely new, as they had already worked with a similar case management innovation (PraCMan) that was included in an earlier version of the CareCockpit software. One GP described this not only as the programme itself, but also as familiarity with a way of working or thinking. In addition, hospitals reported that VESPEERA was easier to integrate if it was compatible with their current workflows, or the opposite: ‘Well! Not to integrate, because integration would have meant replacing one process with another. In this respect, such pilot projects are always extra.’ (hospital management).

In some cases, one has to rely on the cooperation of others to be fall into a new routine. This includes leadership: if a GP does not insist on implementing the VESPEERA programme, the VERAHs implementing it will not either. External pressure or expectations can also encourage implementation, for example when patients demand participation in the VESPEERA programme.

One participant described that VESPEERA was intuitive and therefore used rather unconsciously. However, not all participants felt this way, others rather saw the new innovation as a burden in daily practice. Therefore, repeated training and exercises are needed, as well as a certain regularity in the use of the innovation. This regularity could be more easily ensured if the VESPEERA programme were applicable to a larger number of patients, for example by involving more health insurance companies. On the other hand, other participants described that it is too much of an additional time burden for them if the has to be repeated too often. In general, people had to invest and become active to become more familiar with the VESPEERA programme: ‘[…] but then it's like with everything. You’ve done it once and then it's gone. Then you have to work through it again, ah what was that again, […] and then you start and click through, and it's just very hard at the beginning.’ (GP). This involves additional effort and sufficient resources (time, staff) especially in the early phases of implementation: ‘Well it’s definitely an additional effort, but that’s true for almost all new things that are introduced - until a benefit becomes apparent.’ (hospital management). This also includes a high turnover of staff, which makes implementation difficult. In an environment with a very high workload and stressful daily practice routine, as is the case in hospitals and general practices, it is particularly difficult to make extra efforts: ‘Because, you see, that runs alongside the daily practice routine and now the wave of colds has started and we just have to squeeze them in somewhere all the time.’ (GP).

Moreover, this process takes time. Many participants described this process of training and gaining experience as essential for ‘internalising’ (VERAH) the VESPEERA programme so that it becomes an unconscious behaviour. They aim to act in a way that does not require cognitive and active thinking. One participant described that ‘it just has to click with me’ (VERAH) and she does not have to think about it.

Once individuals have made this step and fall into a new routine, there are contextual factors that make them stay in that routine and prevent them of falling back into old habits. The most important aspects mentioned by participants were that they derive a significant benefit from the VESPEERA programme and that it must be a perceived improvement compared to the previous routine, either for themselves (e.g. appreciation by supervisors), for the organisation or for the patient (e.g. patients report being grateful and satisfied). These benefits must be immediate. However, participants pointed out that some of the benefits show up later and are therefore difficult to assess, such as a possible reduction in readmissions. Doubts about the benefits can hinder persistence in the new routine.

### Reflection: the participants’ conclusion

Some of the participants summarised their experiences with the implementation of the VESPEERA programme. Hospital managers were initially optimistic about the implementation of the programme, had plans to monitor the number of VESPEERA patients and share experiences of implementing the programme, and were eventually disappointed that they were not able to care for VESPEERA patients: “It was very frustrating […] They (the people who were involved) saw the time resources they had invested upfront wasted.’ (hospital management). Other participants from general practices mentioned that once a decision to participate is made, it is first put into action. In doing so, they may find that ‘it's nonsense or it doesn't work’ (VERAH) or that they feel that’ it’s just put into this administrative vat and I think that the human aspect of it gets a bit lost.’ (VERAH). If this is the case, before they discard their original decision to participate, they need to justify their views well to their supervisors. In the case of VESPEERA, this general practice decided to make sacrifices and implement only parts of the programme.

All in all, regardless of the stage of implementation and despite the failures to introduce VESPEERA, many participants were convinced of its benefits and wished for a rollout, as indicated by participants from general practices:

’I didn't think the VESPEERA was that bad. I think it's a very, very good thing, but it's just a shame that it doesn't reach everyone.’ (VERAH).

and from hospitals:

Interviewer: ‘Can you imagine VESPEERA being implemented in usual care at some point?’.

Participant: ‘– From the idea yes, – from the implementation I think it will depend on the acceptance of this project and thus also on the implementation, how it is communicated. […] Yes, so if it is clear that both general practice and hospitals have a win–win situation, even if you have to keep some administrative things and follow a certain protocol, I think it is a good idea, could also imagine that there is a gain because it is similar to treatment pathways or standardisation. If it applies to a large proportion of patients and is applicable, then it becomes routine relatively quickly.’ (nurse).

## Discussion

From this study of the process of implementation of VESPEERA, it became apparent that individuals and organisations were at different stages of the described process from the previous to the new routine. At the different stages, different determinants affected whether it was decided to implement the programme, whether and to what extent it was actually implemented, and whether it continued to be applied.

The VESPEERA programme was mostly perceived positively and participants were convinced of its benefits. Nevertheless, there were factors that hindered the implementation of the programme. The analysis showed that organisational factors are important for implementation. In general, the majority of discharge management improvement programmes focus on hospital-based interventions [3–5]. In our study, hospitals and general practices were involved. Organisational structures and characteristics, including hierarchy, leadership, and the role of middle management, are very different in hospitals and general practices. Thus, the inclusion of staff from both types of organisations allowed for a variety of perspectives and showed that different determinants were relevant for implementation depending on the stage the participants were in at the time of the interview.

### Organisational factors associated with implementation of the VESPEERA programme

A major theme that emerged in participants’ statements on the determinants for the adoption of the VESPEERA programme concerns the impact of organisational factors such as organisational structures, hierarchy and leadership. Hospitals and general practices not only differed in terms of these characteristics, but there were also differences between hospitals, depending mainly on their size (small vs. large, e.g. university hospitals). We hypothesise that one major issue is the distinction between the parallelism of decision-making power and executive power. Factors related to the hierarchical level of the person making the decision to participate in the VESPEERA programme seemed to affect which stage of the implementation process one could reach. There are different hierarchical levels in general practices and hospitals. In smaller organisations such as general practices, those persons who made the decision for participation were mostly those who then applied the intervention, whereas in larger organisations such as hospitals, hierarchy seemed to be more important and there seemed to be a more strict separation between frontline workers and decision makers. This is consistent with the findings of Innis et al. [26], who found that smaller hospitals were more likely to adopt evidence-based discharge practices. The authors explain that smaller hospitals may benefit from fewer levels of management and consequently more direct communication between staff when it comes to implementing evidence-based practices [26].

The role of middle management in the success of adopting and implementing new practices has been widely studied. It is believed that middle managers can facilitate implementation [27] by taking on the role of a champion and promoting a new practice to frontline workers [28, 29]. In our study, there were strong leaders among middle managers, such as discharge managers or case managers, who were appointed project managers, but still implementation was not successful in hospitals. One possible explanation is that the responsibility lay with middle managers, but the VESPEERA components were to be applied by frontline workers. Sometimes they were named, in other cases groups of people rather than a single person were responsible. Another reason, supported by the findings of Chuang et al. [30], could be that any potential benefit expected in our study in terms of reduced readmission rates was easier to anticipate by middle management. However, frontline workers may not have been able to perceive an immediate change patients’ health status when implementing the intervention components and were therefore less committed to its implementation.

### Falling into and staying in the new routine

A theme that emerged in many of the interviews is the importance of routines in implementing change and the factors that influence whether a new routine can be established once the decision to participate in the VESPEERA programme has been made. The participants in our study described the early stages of implementation as rather stressful, as they did not yet have a new routine. In this stage, the new routine is seen only as an additional burden (in terms of resources, time, personnel) for the organisation or its staff without financial balance [31]. In their descriptions, they described the presence of a routine as positive and seemed to prefer the application of the innovation when the new routine developed, as it was impulsive and instinctive and thus required less conscious effort. Veinot et al. [32] summarise that routines contribute to knowledge management, reduce uncertainty and thus increasie organisational capability. Activities become routines when they are recurring events. The authors add that routines require a clear distribution of roles as well as stable and standardised processes. They conclude that for organisational change to occur, previous routines must be proactively deconstructed in order to integrate new routines [32]. In relation to our findings, a vicious circle can be observed: A small sample size could explain that it was difficult to establish the new routine and the lack of an established routine (and in some cases, the lack of a responsible person who allowed the routine to emerge) possibly made it difficult to apply the innovation to more patients. However, some participants described that the establishment of a new routine was not possible due to the daily workload, as they could not focus on the innovation, but had to ‘keep putting it in between’ (HA-A13).

The participants’ statements showed that at any point in the process of falling and staying in the new routine, one can conclude that the new routine is not better that the previous one. This may be the case if the daily business in hospitals and general practices does not allow for the emergence of new routines in the implementation process or if, after testing the new routine, critical reflection shows no benefit of the new routine compared to the previous routine. This leads to a loop back to the previous routine: individuals return to their previous routine and stick to it for the same reason why they/ others did not break the old routine in the first place. In the statements of the interviewees, it could be observed that they seemed to be in different stages of this implementation process. Some seemed to have gone through all four of the described stages and were able to reflect on the whole implementation process, others described the process only up to the adoption stage.

### Strengths and limitations

This study has some limitations, such as two types of selection bias. First, the general practices that participated in the VESPEERA project were mostly motivated but already had a good performance in terms of care transitions (based on their own reports). As is often the case in health care, the innovation did not reach those who could most benefit from it, which may also have affected the results of the outcomes evaluation [19]. Second, interview participants may have been more motivated than those who did not participate. All of the hospitals and many of the general practices had few to no patients receiving the innovation. Therefore, few participants were able to talk about all stages of implementation.

## Conclusion

This study provided insights into the factors that determine the adoption, implementation and reflection upon sustaining of a complex intervention to improve care transitions. The individuals and different organisations (i.e. hospitals and general practices) participating in the VESPEERA programme were at different stages of a process from the previous to the new routine. These stages were characterised by different determinants of implementation. Little distinction between decision-making power and executive power seemed to be helpful in escalating to the next stage. Available resources supported the development of a new routine, which was seen as beneficial for the implementation of the innovation.

## Data Availability

The datasets generated and/or analysed during the current study are not publicly available due to them containing information that could compromise research participant privacy but are available from the corresponding author on reasonable request.

## Abbreviations

AOK: Allgemeine Ortskrankenkasse, large German sickness fund
CFIR: Consolidated Framework for Implementation Research
GP: General practitioner
VERAH: Care Assistant in General Practice (Versorgungsassistentin in der Hausarztpraxis)
VESPEERA: Improving care across sectors: An admission and discharge model in general practice and hospitals (German: Versorgungskontinuität sichern: Patientenorientiertes Einweisungs- und Entlassmanagement in Hausarztpraxen und Krankenhäusern)
